# The Story of the Dopamine Transporter PET Tracer LBT-999: From Conception to Clinical Use

**DOI:** 10.3389/fmed.2019.00090

**Published:** 2019-05-03

**Authors:** Sylvie Chalon, Johnny Vercouillie, Pierre Payoux, Jean-Bernard Deloye, Cécile Malherbe, Florence Le Jeune, Nicolas Arlicot, Anne-Sophie Salabert, Denis Guilloteau, Patrick Emond, Maria-Joao Ribeiro

**Affiliations:** ^1^UMR 1253, iBrain, Université de Tours, Inserm, Tours, France; ^2^Inserm CIC 1415, University Hospital, Tours, France; ^3^ToNIC, Toulouse NeuroImaging Center, Université de Toulouse, Inserm, UPS, Toulouse, France; ^4^University Hospital, Nuclear Medicine Unit, Toulouse, France; ^5^Zionexa, 42 avenue de la Grande Armée, Paris, France; ^6^University of Rennes 1, Rennes, France; ^7^Department of Nuclear Medicine, Centre Eugène Marquis, Rennes, France; ^8^CHRU Tours, Tours, France

**Keywords:** PET, dopaminergic neuron, Parkinson's disease, radiopharmaceutical, basal ganglia

## Abstract

The membrane dopamine transporter (DAT) is involved in a number of brain disorders and its exploration by positron emission tomography (PET) imaging is highly relevant for the early and differential diagnosis, follow-up and treatment assessment of these diseases. A number of carbon-11 and fluor-18 labeled tracers are to date available for this aim, the majority of them being derived from the chemical structure of cocaine. The development of such a tracer, from its conception to its use, is a long process, the expected result being to obtain the best radiopharmaceutical adapted for clinical protocols. In this context, the cocaine derivative *(E)-N*-(4-fluorobut-2-enyl)2β-carbomethoxy-3β-(4′-tolyl)nortropane, or LBT-999, has passed all the required stages of the development that makes it now a highly relevant imaging tool, particularly in the context of Parkinson's disease. This review describes the different steps of the development of LBT-999 which initially came from its non-fluorinated derivative (*E*)-*N*-(3-iodoprop-2-enyl)-2-carbomethoxy-3-(4-methylphenyl) nortropane, or PE2I, because of its high promising properties. [^18^F]LBT-999 has been extensively characterized in rodent and non-human primate models, in which it demonstrated its capability to explore *in vivo* the DAT localized at the dopaminergic nerve endings as well as at the mesencephalic cell bodies, in physiological conditions. In lesion-induced rat models of Parkinson's disease, [^18^F]LBT-999 was able to precisely quantify *in vivo* the dopaminergic neuron loss, and to assess the beneficial effects of therapeutic approaches such as pharmacological treatment and cell transplantation. Finally recent clinical data demonstrated the efficiency of [^18^F]LBT-999 in the diagnosis of Parkinson's disease.

## *In vivo* Imaging of the DAT: a Highly Potent Tool for Brain Disorders

The dopaminergic neurotransmission is strongly involved in the regulation of multiple brain functions such as locomotion, cognition and reward, and then plays a major role in a great number of brain disorders such as Parkinson's disease (PD) (1) but also several neuropsychiatric disorders (2). In this context, *in vivo* exploration of this system through molecular imaging methods is a real added value for the diagnosis, follow-up, and treatment of such disorders. Several molecular targets of the dopaminergic neurotransmission can be explored *in vivo*, at both the pre- and post-synaptic level. These explorations require the use of specific radiotracers able to bind specifically to each target and then to quantify it as accurately as possible. For this aim a high number of tracers have been developed, either labeled with γ emitters such as ^123^I or ^99m^Tc for single photon emission tomography (SPECT), or with β+ emitters such as ^11^C or ^18^F for positron emission tomography (PET). Several tracers are yet available for the different types of post-synaptic dopaminergic receptors (3). Regarding pre-synaptic dopaminergic neurons, SPECT and/or PET exploration of three main molecular targets are to date available. The 6-[^18^F]-fluoro-L-dopa or [^18^F]DOPA uptake, which reflects both the conversion of Dopa into dopamine (DA) and the storage of DA into synaptic vesicles, has been the first gold standard tool (4). Besides, the vesicular monoamine transporter 2 (VMAT2) and the membrane dopamine transporter (DAT) can also be explored. The respective advantages and drawbacks related to imaging these different pre-synaptic molecular targets have mainly been compared in the context of PD, and prominent conclusions are summarized in Table 1.

**Table 1:** Presynaptic molecular PET imaging targets of the dopaminergic neurotransmission.

**Molecular target**	**Examples of tracer**	**Advantages**	**Drawbacks**	**References**
DOPA decarboxylase	[^18^F]F-DOPA	– Distinguishes patients with advanced PD from patients with *de novo* PD	– Reflects both the conversion of Dopa into DA and pre-synaptic storage of DA – Possible under-estimation of DA neurons loss in *de novo* PD patients due to an up-regulation of DA synthesis	(5–7)
Vesicular monoamine transporter 2 (VMAT2)	[^11^C]DTBZ [^18^F]AV-133	– Detects early PD vs. healthy controls – Improves diagnostic accuracy in clinically uncertain parkinsonian syndrome	– Present on all monoaminergic neurons	(8–10)
Membrane dopamine transporter (DAT)	[^18^F]FP-CIT [^11^C]PE2I [^18^F]FE-PE2I	– Distinguishes patients with advanced PD from patients with *de novo* PD – Greater sensitivity than F-DOPA for detecting motor severity in PD – Identification of patients at risks for developing PSP or FTD	– Possible over-estimation of DA neurons loss due to a down-regulation of the DAT	(5, 11, 12)

*AV-133, fluoropropyldihydrotetrabenazine; DA, dopamine; F-DOPA, 6-fluoro-dopa; DTBZ, dihydrotetrabenazine, FDT, frontotemporal dementia; PSP, progressive supranuclear palsy*.

For a long time, the DAT has been identified as a target of choice because its localization makes it a marker of neuron integrity and density, and also because it is a key-actor in the regulation of synaptic dopamine levels (13). A high number of SPECT and PET tracers have been developed for DAT imaging. In all cases, they were derived from known ligands of the DAT, and most of them from the tropane structure characteristic of cocaine (14). The first of these tracers which demonstrated its potency in the field of PD using SPECT imaging was the 2β-carbomethoxy-3β-(4-iodophenyl)tropane (β-CIT) (15), which bound to the DAT with a high affinity (around 3 nM) and accumulated significantly in dopaminergic brain areas when labeled with iodine-123. Although β-CIT demonstrated its usefulness for the detection of DAT loss in PD, it had several drawbacks such as a similar affinity for the dopamine and serotonin transporters (16), a poor signal/noise ratio and an *in vivo* kinetics requiring as long as 24 h to reach equilibrium state allowing the DAT quantification in the striatum (17).

A number of new β-CIT derivatives were then proposed to overcome these weaknesses. Among them, the N-(3-iodopro-2*E*-enyl)-2β-carbomethoxy-3β-(4-methylphenyl)nortropane (PE2I) is structurally characterized by the presence of a methyl group on the phenyl ring of the β-CIT structure instead of an iodine, and a 3-iodopro-2E-enyl group at the tropane nitrogen instead of a methyl carried by β-CIT (18). These chemical modifications have led to a significant improvement in the pharmacological profile of this ligand (19, 20), showing a high selectivity for the DAT toward the serotonin transporter (SERT). The high affinity and selectivity made PE2I a highly potent tracer to image the DAT *in vivo* either by SPECT when labeled with ^123^I and by PET when labeled with ^11^C. In this context, [^123^I]PE2I demonstrated its usefulness for the differential diagnosis between patients suffering from PD and atypical parkinsonian syndromes without degeneration of striatal dopaminergic nerve endings (21). The PET imaging with [^11^C]PE2I has also been successfully used in this same disease (11, 22) but also in schizophrenia (23, 24), attention deficit / hyperactivity disorders (25) and more recently in the exploration of the reward dopaminergic pathway (26).

## Development of LBT-999

**Figure 1:**
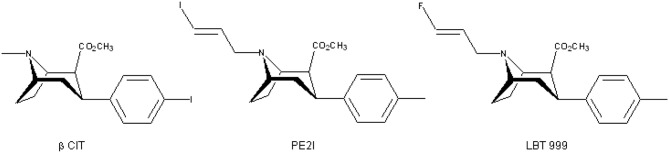
Chemical structures of β-CIT, PE2I, and LBT-999.

Regarding the high potency of binding of PE2I for the DAT and because PET imaging enables *in vivo* exploration at high resolution and high sensitivity, we developed the fluorinated derivative of PE2I, i.e., 8-((E)-4-fluoro-but-2-enyl)-3β-p-tolyl-8-aza-bicyclo[3.2.1]octane-2β-carboxylic acid methyl ester (LBT-999) (Figure 1).

The *in vitro* pharmacological evaluation of LBT-999 demonstrated that its properties was close to that of PE2I, with a good affinity for the DAT (9 nM) and a Ki > 1 μM for different ligands of the serotonin and norepinephrine transporters (27). Firstly, LBT-999 was labeled with carbon-11 (28) by methylation of the acid precursor that can be obtained in an easier way compared to a precursor useable for fluorine labeling. The [^11^C]LBT-999 shown to have a high *in vivo* accumulation in brain areas containing high levels of DAT both in rats and monkeys (27, 28). Based on these results, the development of the radiolabeling with [^18^F] was then realized, first using a two-step methodology (29) followed by a one-step approach (30) required for rapid and reproducible radiofluorination dedicated to preclinical and clinical studies. As for the [^11^C]LBT-999, [^18^F]LBT-999 rapidly, and highly entered the rat brain where its distribution was in agreement with the DAT density. Importantly, 1 h post-injection, the *in vivo* specific binding represented by the ratio of accumulation in the striatum to cerebellum, was 10 times higher for LBT-999 (ratio of 25) (27) compared to that we obtained previously with PE2I in same experimental conditions (31). For LBT as for PE2I, the striatal accumulation at 1 h post-injection was around 70% decreased in the presence of a saturating dose of the DAT inhibitor GBR12909, whereas no significant effect was observed with a pre-injection of paroxetine (SERT ligand) or nisoxetine (NET ligand). In monkey, LBT-999 was also able to bind specifically to the DAT, either labeled with [^11^C] (27) or with [^18^F] (32). This last study demonstrated that LBT was also suitable for DAT exploration in extra-striatal regions, and that the estimated dosimetry was acceptable for human use.

## Preclinical Experiments in Animal Models

**Figure 2:**
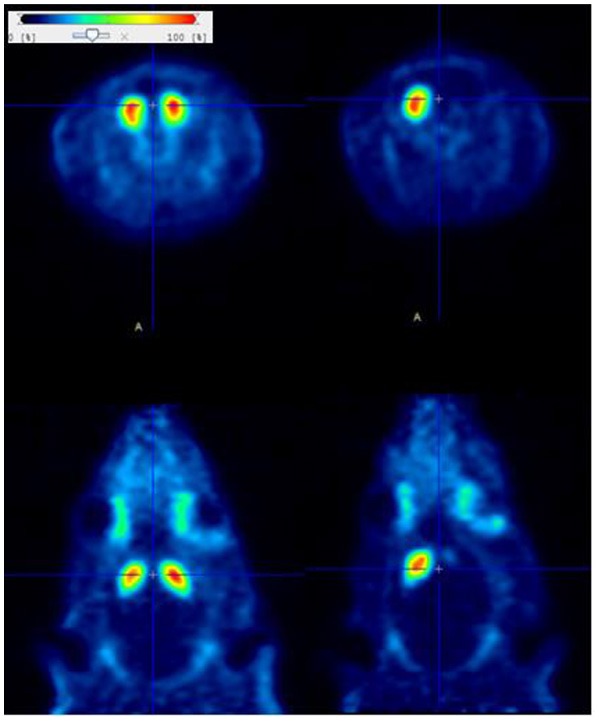
Coronal (upper side) and axial (lower side) PET static images (30–50 min post-injection) obtained with [^18^F]LBT-999 in a normal rat (left) and in a rat lesioned with 6-OHDA in the right striatum. The quantitative analysis revealed a decreased of 70% in the tracer accumulation in the lesioned vs. intact striatum.

As the final aim of the development of a new PET tracer is its use for human health improvement, it is of high value to explore the properties of such a candidate tracer in animal models of human diseases. For this purpose, we performed in a first step in rats, an extensive test-retest study that demonstrated the ability of [^18^F]LBT-999 to quantify the DAT with high reproducibility (variability of 8–14%) and reliability (intra-class correlation coefficient, ICC, of 0.9) in the striatum, whereas these parameters were less accurate in the substantia nigra, in relation with the small size of this brain structure (33). In a rat model of early PD induced by a moderate unilateral striatal lesion using 6-hydroxydopamine (6-OHDA), we showed that [^18^F]LBT-999 was able to accurately quantify *in vivo* the dopaminergic endings loss (Figure 2), in full agreement with the results obtained by *in vitro* autoradiography with [^125^I]PE2I on brain sections (34).

It was also important to assess the potency of [^18^F]LBT-999 to evaluate the efficacy of various therapeutic approaches aiming at the preservation or replacement of dopaminergic neurons *in vivo* in the rat model of 6-OHDA lesions. This property was demonstrated in the case of a pharmacological therapeutic approach (35) as well as for the graft of human embryonic stem cells-derived midbrain dopaminergic neurons (36). These whole findings provided strong preclinical support for clinical translation of [^18^F]LBT-999.

## The Use of [^18^F]LBT-999 in Human

**Figure 3:**
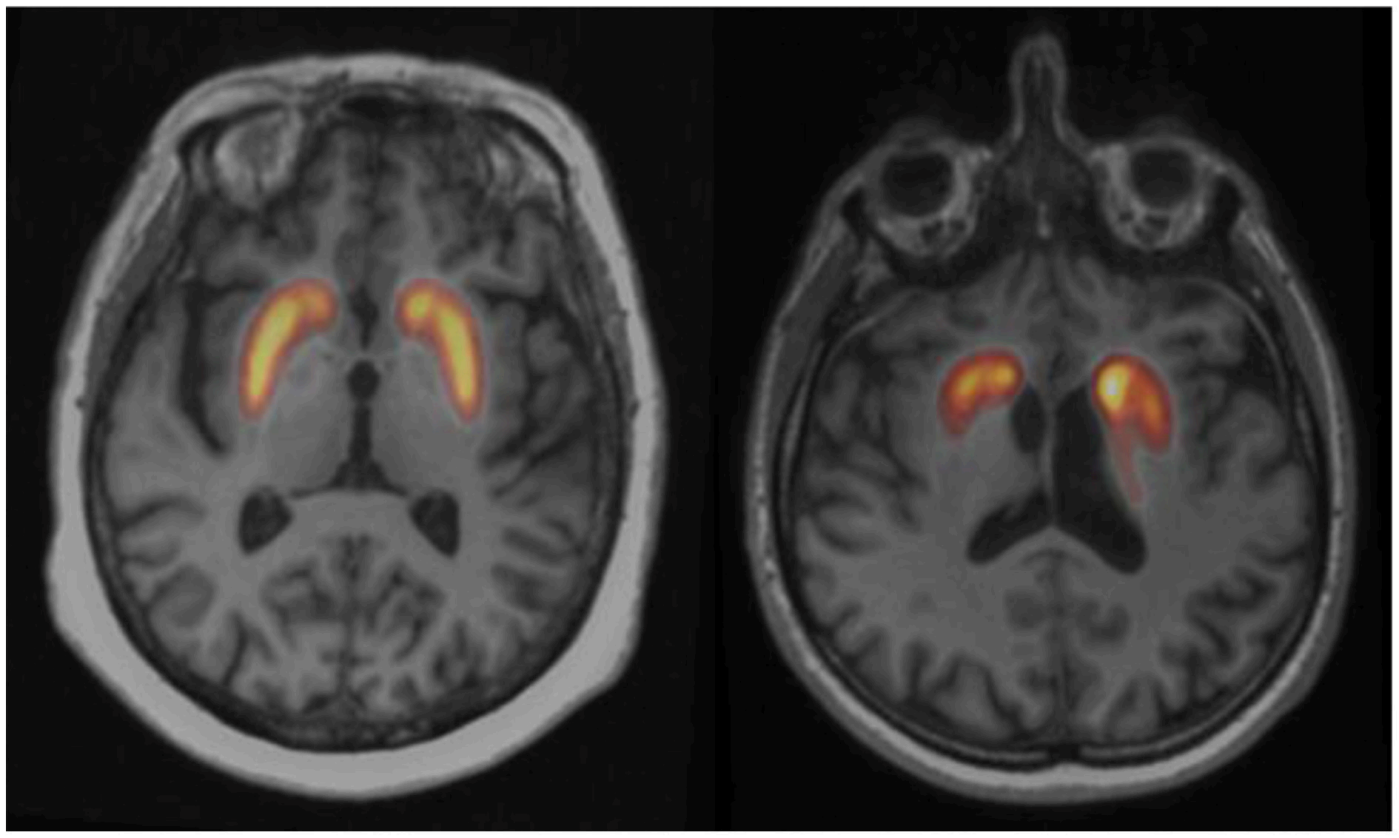
Fusion axial slices between PET and MRI of the [^18^F]LBT-999 uptake at the level of the striatum in a control subject (left) and a drug-naïve patient with early Parkinson disease (right). The radiopharmaceutical uptake is asymmetrically decreased in Parkinson patient.

[^18^F]LBT-999 has recently been evaluated in clinical setting (37, 38). Preliminary results on a small sample of 6 subjects with early Parkinson's disease and 8 healthy controls demonstrated that injection of [^18^F]LBT-999 is feasible and pharmacologically safe. [^18^F]LBT-999 distribution was consistent with DAT density in human brain and PET images in both caudate and putamen nuclei indicate that this tracer may successfully differentiate the two groups of subjects (Figure 3). On the basis of these initial findings, [^18^F]LBT-999 might be a suitable radiopharmaceutical for PET assessment of DAT in future clinical studies.

## Conclusions

After the identification of a brain molecular target whose PET exploration would be crucial for improvement of the diagnosis and/or treatment of a particular disease, it is a long way to make available an optimal radiotracer. A very high number of tracers have been developed as potential DAT imaging agents, the most promising being based on the tropane scaffold derived from the structure of cocaine. Several SPECT compounds are used in clinical protocols, such as ^99m^Tc-TRODAT (39) and [123I]FP-CIT (40). However, they suffer from many disadvantages such as poor sensitivity, spatial resolution, and slow kinetic uptake, and PET ligands should be a good alternative. We described in this paper the development of one of these tracers, [^18^F]LBT-999, which has the particularity to be highly specific for its target, and which is now ready to be used for clinical purpose.

## Author Contributions

All authors listed have made a substantial, direct and intellectual contribution to the work, and approved it for publication.

### Conflict of Interest Statement

J-BD was employed by company Zionexa, Paris, France. The remaining authors declare that the research was conducted in the absence of any commercial or financial relationships that could be construed as a potential conflict of interest.
